# Partial Scanning Transmission Electron Microscopy with Deep Learning

**DOI:** 10.1038/s41598-020-65261-0

**Published:** 2020-05-20

**Authors:** Jeffrey M. Ede, Richard Beanland

**Affiliations:** University of Warwick, Department of Physics, Coventry, CV4 7AL UK

**Keywords:** Imaging techniques, Scientific data, Imaging techniques, Microscopy, Imaging techniques, Microscopy

## Abstract

Compressed sensing algorithms are used to decrease electron microscope scan time and electron beam exposure with minimal information loss. Following successful applications of deep learning to compressed sensing, we have developed a two-stage multiscale generative adversarial neural network to complete realistic 512 × 512 scanning transmission electron micrographs from spiral, jittered gridlike, and other partial scans. For spiral scans and mean squared error based pre-training, this enables electron beam coverage to be decreased by 17.9× with a 3.8% test set root mean squared intensity error, and by 87.0× with a 6.2% error. Our generator networks are trained on partial scans created from a new dataset of 16227 scanning transmission electron micrographs. High performance is achieved with adaptive learning rate clipping of loss spikes and an auxiliary trainer network. Our source code, new dataset, and pre-trained models are publicly available.

## Introduction

Aberration corrected scanning transmission electron microscopy (STEM) can achieve imaging resolutions below 0.1 nm, and locate atom columns with pm precision^1,2^. Nonetheless, the high current density of electron probes produces radiation damage in many materials, limiting the range and type of investigations that can be performed^3,4^. A number of strategies to minimize beam damage have been proposed, including dose fractionation^5^ and a variety of sparse data collection methods^6^. Perhaps the most intensively investigated approach to the latter is sampling a random subset of pixels, followed by reconstruction using an inpainting algorithm^3,6–10^. Poisson random sampling of pixels is optimal for reconstruction by compressed sensing algorithms^11^. However, random sampling exceeds the design parameters of standard electron beam deflection systems, and can only be performed by collecting data slowly^12,13^, or with the addition of a fast deflection or blanking system^3,14^.

Sparse data collection methods that are more compatible with conventional beam deflection systems have also been investigated. For example, maintaining a linear fast scan deflection whilst using a widely-spaced slow scan axis with some small random ‘jitter’^9,12^. However, even small jumps in electron beam position can lead to a significant difference between nominal and actual beam positions in a fast scan. Such jumps can be avoided by driving functions with continuous derivatives, such as those for spiral and Lissajous scan paths^3,13,15,16^. Sang^13,16^ considered a variety of scans including Archimedes and Fermat spirals, and scans with constant angular or linear displacements, by driving electron beam deflectors with a field-programmable gate array (FPGA) based system. Spirals with constant angular velocity place the least demand on electron beam deflectors. However, dwell times, and therefore electron dose, decreases with radius. Conversely, spirals created with constant spatial speeds are prone to systematic image distortions due to lags in deflector responses. In practice, fixed doses are preferable as they simplify visual inspection and limit the dose dependence of STEM noise^17^.

Deep learning has a history of successful applications to image infilling, including image completion^18^, irregular gap infilling^19^ and supersampling^20^. This has motivated applications of deep learning to the completion of sparse, or ‘partial’, scans, including supersampling of scanning electron microscopy^21^ (SEM) and STEM images^22,23^. Where pre-trained models are unavailable for transfer learning^24^, artificial neural networks (ANNs) are typically trained, validated and tested with large, carefully partitioned machine learning datasets^25,26^ so that they are robust to general use. In practice, this often requires at least a few thousand examples. Indeed, standard machine learning datasets such as CIFAR-10^27,28^, MNIST^29^, and ImageNet^30^ contain tens of thousands or millions of examples. To train an ANN to complete STEM images from partial scans, an ideal dataset might consist of a large number of pairs of partial scans and corresponding high-quality, low noise images, taken with an aberration-corrected STEM. To our knowledge, such a dataset does not exist. As a result, we have collated a new dataset of STEM raster scans from which partial scans can be selected. Selecting partial scans from full scans is less expensive than collecting image pairs, and individual pixels selected from experimental images have realistic noise characteristics.

**Figure 1:**
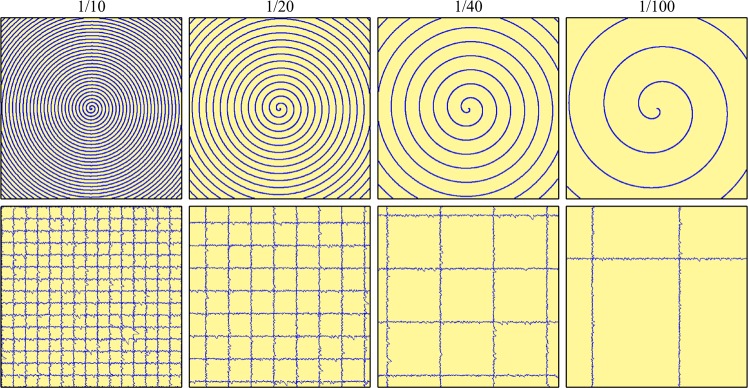
Examples of Archimedes spiral (top) and jittered gridlike (bottom) 512 × 512 partial scan paths for 1/10, 1/20, 1/40, and 1/100 px coverage.

Examples of spiral and jittered gridlike partial scans investigated in this paper are shown in Fig. 1. Continuous spiral scan paths that extend to image corners cannot be created by conventional scan systems without going over image edges. However, such a spiral can be cropped from a spiral with radius at least 2^−1/2^ times the minimum image side, at the cost of increased scan time and electron beam damage to the surrounding material. We use Archimedes spirals, where $r\propto \theta$, and *r* and *θ* are polar radius and angle coordinates, as these spirals have the most uniform spatial coverage. Jittered gridlike scans would also be difficult to produce with a conventional system, which would suffer variations in dose and distortions due to limited beam deflector response. Nevertheless, these idealized scan paths serve as useful inputs to demonstrate the capabilities of our approach. We expect that other scan paths could be used with similar results.

We fine-tune our ANNs as part of generative adversarial networks^31^ (GANs) to complete realistic images from partial scans. A GAN consists of sets of generators and discriminators that play an adversarial game. Generators learn to produce outputs that look realistic to discriminators, while discriminators learn to distinguish between real and generated examples. Limitedly, discriminators only assess whether outputs look realistic; not if they are correct. This can result in a neural network only generating a subset of outputs, referred to as mode collapse^32^. To counter this issue, generator learning can be conditioned on an additional distance between generated and true images^33^. Meaningful distances can be hand-crafted or learned automatically by considering differences between features imagined by discriminators for real and generated images^34,35^.

## Training

In this section we introduce a new STEM images dataset for machine learning, describe how partial scans were selected from images in our data pipeline, and outline ANN architecture and learning policy. Detailed ANN architecture, learning policy, and experiments are provided as Supplementary Information, and source code is available^36^.

### Data pipeline

To create partial scan examples, we collated a new dataset containing 16227 32-bit floating point STEM images collected with a JEOL ARM200F atomic resolution electron microscope. Individual micrographs were saved to University of Warwick data servers by dozens of scientists working on hundreds of projects as Gatan Microscopy Suite^37^ generated dm3 or dm4 files. As a result, our dataset has a diverse constitution. Atom columns are visible in two-thirds of STEM images, with most signals imaged at several times their Nyquist rates^38^, and similar proportions of images are bright and dark field. The other third of images are at magnifications too low for atomic resolution, or are of amorphous materials. Importantly, our dataset contains noisy images, incomplete scans and other low-quality images that would not normally be published. This ensures that ANNs trained on our dataset are robust to general use. The Digital Micrograph image format is rarely used outside the microscopy community. As a result, data has been transferred to the widely supported TIFF^39^ file format in our publicly available dataset^40,41^.

Micrographs were split into 12170 training, 1622 validation, and 2435 test set examples. Each subset was collected by a different subset of scientists and has different characteristics. As a result, unseen validation and test sets can be used to quantify the ability of a trained network to generalize. To reduce data read times, each micrograph was split into non-overlapping 512 × 512 sub-images, referred to as ‘crops’, producing 110933 training, 21259 validation and 28877 test set crops. For convenience, our crops dataset is also available^40,41^. Each crop, $I$, was processed in our data pipeline by replacing non-finite electron counts, i.e. NaN and ±$\infty$, with zeros. Crops were then linearly transformed to have intensities ${I}_{{\rm{N}}}\in [\,-\,1,1]$, except for uniform crops satisfying ${\rm{\max }}(I)-\,{\rm{\min }}(I) < {10}^{-6}$ where we set ${I}_{{\rm{N}}}=0$ everywhere. Finally, each crop was subject to a random combination of flips and 90° rotations to augment the dataset by a factor of eight.

Partial scans, ${I}_{{\rm{scan}}}$, were selected from raster scan crops, ${I}_{{\rm{N}}}$, by multiplication with a binary mask ${\Phi }_{{\rm{path}}}$,

1 $${I}_{{\rm{scan}}}={\Phi }_{{\rm{path}}}{I}_{{\rm{N}}},$$

where ${\varPhi }_{{\rm{path}}}=1$ on a scan path, and ${\varPhi }_{{\rm{path}}}=0$ otherwise. Raster scans are sampled at a rectangular lattice of discrete locations, so a subset of raster scan pixels are experimental measurements. In addition, although electron probe position error characteristics may differ for partial and raster scans, typical position errors are small^42,43^. As a result, we expect that partial scans selected from raster scans with binary masks are realistic.

We also selected partial scans with blurred masks to simulate varying dwell times and noise characteristics. These difficulties are encountered in incoherent STEM^44,45^, where STEM illumination is detected by a transmission electron microscopy (TEM) camera. For simplicity, we created non-physical noise by multiplying ${I}_{{\rm{scan}}}$ with $\eta ({\Phi }_{{\rm{path}}})={\Phi }_{{\rm{path}}}+(1-{\Phi }_{{\rm{path}}})U$, where *U* is a uniform random variate distributed in [0, 2). ANNs are able to generalize^46,47^, so we expect similar results for other noise characteristics. A binary mask, with values in $\{0,1\}$, is a special case where no noise is applied i.e. $\eta (1)=1$, and ${\varPhi }_{{\rm{path}}}=0$ is not traversed. Performance is reported for both binary and blurred masks.

The noise characteristics in our new STEM images dataset vary. This is problematic for mean squared error (MSE) based ANN training losses, as differences are higher for crops with higher noise. In effect, this would increase the importance of noisy images in the dataset, even if they are not more representative. Although adaptive ANN optimizers that divide parameter learning rates by gradient sizes^48^ can partially mitigate weighting by varying noise levels, this restricts training to a batch size of 1 and limits momentum. Consequently, we low-passed filtered ground truth images, ${I}_{N}$, to ${I}_{{\rm{blur}}}$ by a 5 × 5 symmetric Gaussian kernel with a 2.5 px standard deviation, to calculate MSEs for ANN outputs.

### Network architecture

**Figure 2:**
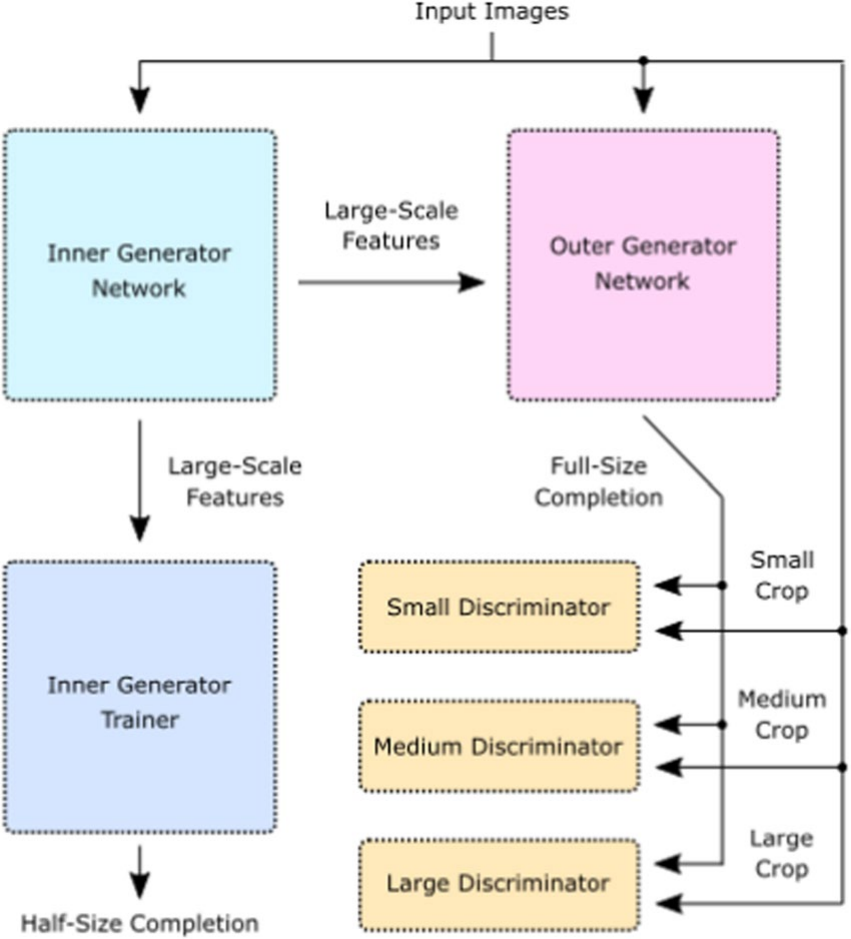
Simplified multiscale generative adversarial network. An inner generator produces large-scale features from inputs. These are mapped to half-size completions by a trainer network and recombined with the input to generate full-size completions by an outer generator. Multiple discriminators assess multiscale crops from input images and full-size completions. This figure was created with Inkscape^83^.

To generate realistic images, we developed a multiscale conditional GAN with TensorFlow^49^. Our network can be partitioned into the six convolutional^50,51^ subnetworks shown in Fig. 2: an inner generator, ${G}_{{\rm{inner}}}$, outer generator, ${G}_{{\rm{outer}}}$, inner generator trainer, $T$, and small, medium and large scale discriminators, ${D}_{1}$, ${D}_{2}$ and ${D}_{3}$. We refer to the compound network $G({I}_{{\rm{scan}}})={G}_{{\rm{outer}}}({G}_{{\rm{inner}}}({I}_{{\rm{scan}}}),{I}_{{\rm{scan}}})$ as the generator, and to *D* = {*D*_1_, *D*_2_, *D*_3_} as the multiscale discriminator. The generator is the only network needed for inference.

Following recent work on high-resolution conditional GANs^34^, we use two generator subnetworks. The inner generator produces large scale features from partial scans bilinearly downsampled from 512 × 512 to 256 × 256. These features are then combined with inputs embedded by the outer generator to output full-size completions. Following Inception^52,53^, we introduce an auxiliary trainer network that cooperates with the inner generator to output 256 × 256 completions. This acts as a regularization mechanism, and provides a more direct path for gradients to backpropagate to the inner generator. To more efficiently utilize initial generator convolutions, partial scans selected with a binary mask are nearest neighbour infilled before being input to the generator.

Multiscale discriminators examine real and generated STEM images to predict whether they are real or generated, adapting to the generator as it learns. Each discriminator assesses different-sized crops selected from 512 × 512 images, with sizes 70 × 70, 140 × 140 or 280 × 280. After selection, crops are bilinearly downsampled to 70 × 70 before discriminator convolutions. Typically, discriminators are applied at fractions of the full image size^34^ e.g. 512/2^2^, 512/2^1^ and 512/2^0^. However, we found that discriminators that downsample large fields of view to 70 × 70 are less sensitive to high-frequency STEM noise characteristics. Processing fixed size image regions with multiple discriminators has been proposed^54^ to decrease computation for large images, and extended to multiple region sizes^34^. However, applying discriminators to arrays of non-overlapping image patches^55^ results in periodic artefacts^34^ that are often corrected by larger-scale discriminators. To avoid these artefacts and reduce computation, we apply discriminators to randomly selected regions at each spatial scale.

### Learning policy

Training has two halves. In the non-adversarial first half, the generator and auxiliary trainer cooperate to minimize mean squared errors (MSEs). This is followed by an optional second half of training, where the generator is fine-tuned as part of a GAN to produce realistic images. Our ANNs are trained by ADAM^56^ optimized stochastic gradient descent^48,57^ for up to 2 × 10^6^ iterations, which takes a few days with an Nvidia GTX 1080 Ti GPU and an i7-6700 CPU. The objectives of each ANN are codified by their loss functions.

In the non-adversarial first half of training, the generator, $G$, learns to minimize the MSE based loss

2 $${L}_{{\rm{MSE}}}={\rm{ALRC}}({\lambda }_{{\rm{cond}}}{\rm{MSE}}(G({I}_{{\rm{scan}}}),{I}_{{\rm{blur}}})),$$

where ${\lambda }_{{\rm{cond}}}=200$, and adaptive learning rate clipping^58^ (ALRC) is important to prevent high loss spikes from destabilizing learning. Experiments with and without ALRC are in Supplementary Information. To compensate for varying noise levels, ground truth images were blurred by a 5 × 5 symmetric Gaussian kernel with a 2.5 px standard deviation. In addition, the inner generator, ${G}_{{\rm{inner}}}$, cooperates with the auxiliary trainer, $T$, to minimize

3 $${L}_{{\rm{aux}}}={\rm{ALRC}}({\lambda }_{{\rm{trainer}}}{\rm{MSE}}(T({G}_{{\rm{inner}}}({I}_{{\rm{scan}}}^{{\rm{half}}}))),{I}_{{\rm{blur}}}^{{\rm{half}}}),$$

where ${\lambda }_{{\rm{trainer}}}=200$, and ${I}_{{\rm{scan}}}^{{\rm{half}}}$ and ${I}_{{\rm{blur}}}^{{\rm{half}}}$ are 256 × 256 inputs bilinearly downsampled from ${I}_{{\rm{scan}}}$ and ${I}_{{\rm{blur}}}$, respectively.

In the optional adversarial second half of training, we use $N=3$ discriminator scales with numbers, ${N}_{1}$, ${N}_{2}$ and ${N}_{3}$, of discriminators, ${D}_{1}$, ${D}_{2}$ and ${D}_{3}$, respectively. There many popular GAN loss functions and regularization mechanisms^59,60^. In this paper, we use spectral normalization^61^ with squared difference losses^62^ for the discriminators,

4 $${L}_{D}=\frac{1}{N}\,\mathop{\sum }\limits_{i=1}^{N}\,\frac{1}{{N}_{i}}[{D}_{i}{(G({I}_{{\rm{scan}}}))}^{2}+{({D}_{i}({I}_{N})-\mathrm{1)}}^{2}],$$

where discriminators try to predict 1 for real images and 0 for generated images. We found that ${N}_{1}={N}_{2}={N}_{3}=1$ is sufficient to train the generator to produce realistic images. However, higher performance might be achieved with more discriminators e.g. 2 large, 8 medium and 32 small discriminators. The generator learns to minimize the adversarial squared difference loss,

5 $${L}_{{\rm{adv}}}=\frac{1}{N}\,\mathop{\sum }\limits_{i=1}^{N}\,\frac{1}{{N}_{i}}{D}_{i}{(G({I}_{{\rm{scan}}})-\mathrm{1)}}^{2},$$

by outputting completions that look realistic to discriminators.

Discriminators only assess the realism of generated images; not if they are correct. To the lift degeneracy and prevent mode collapse, we condition adversarial training on non-adversarial losses. The total generator loss is

6 $${L}_{G}={\lambda }_{{\rm{adv}}}{L}_{{\rm{adv}}}+{L}_{{\rm{MSE}}}+{\lambda }_{{\rm{aux}}}{L}_{{\rm{aux}}},$$

where we found that ${\lambda }_{{\rm{aux}}}=1$ and ${\lambda }_{{\rm{adv}}}=5$ is effective. We also tried conditioning the second half of training on differences between discriminator imagination^34,35^. However, we found that MSE guidance converges to slightly lower MSEs and similar structural similarity indexes^63^ for STEM images.

## Performance

**Figure 3:**
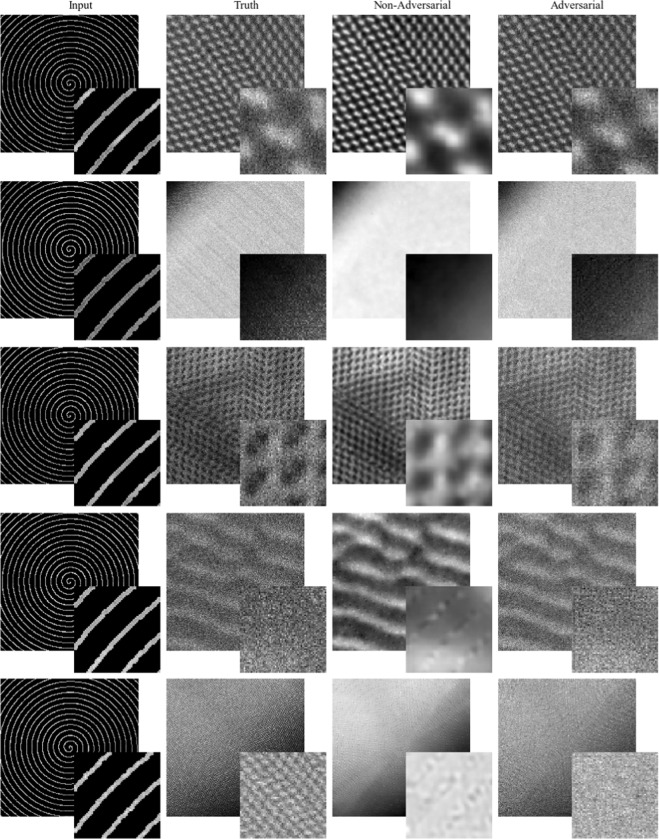
Adversarial and non-adversarial completions for 512 × 512 test set 1/20 px coverage blurred spiral scan inputs. Adversarial completions have realistic noise characteristics and structure whereas non-adversarial completions are blurry. The bottom row shows a failure case where detail is too fine for the generator to resolve. Enlarged 64 × 64 regions from the top left of each image are inset to ease comparison, and the bottom two rows show non-adversarial generators outputting more detailed features nearer scan paths.

To showcase ANN performance, example applications of adversarial and non-adversarial generators to 1/20 px coverage partial STEM completion are shown in Fig. 3. Adversarial completions have more realistic high-frequency spatial information and structure, and are less blurry than non-adversarial completions. Systematic spatial variation is also less noticeable for adversarial completions. For example, higher detail along spiral paths, where errors are lower, can be seen in the bottom two rows of Fig. 3 for non-adversarial completions. Inference only requires a generator, so inference times are the same for adversarial and non-adversarial completions. Single image inference time during training is 45 ms with an Nvidia GTX 1080 Ti GPU, which is fast enough for live partial scan completion.

**Figure 4:**
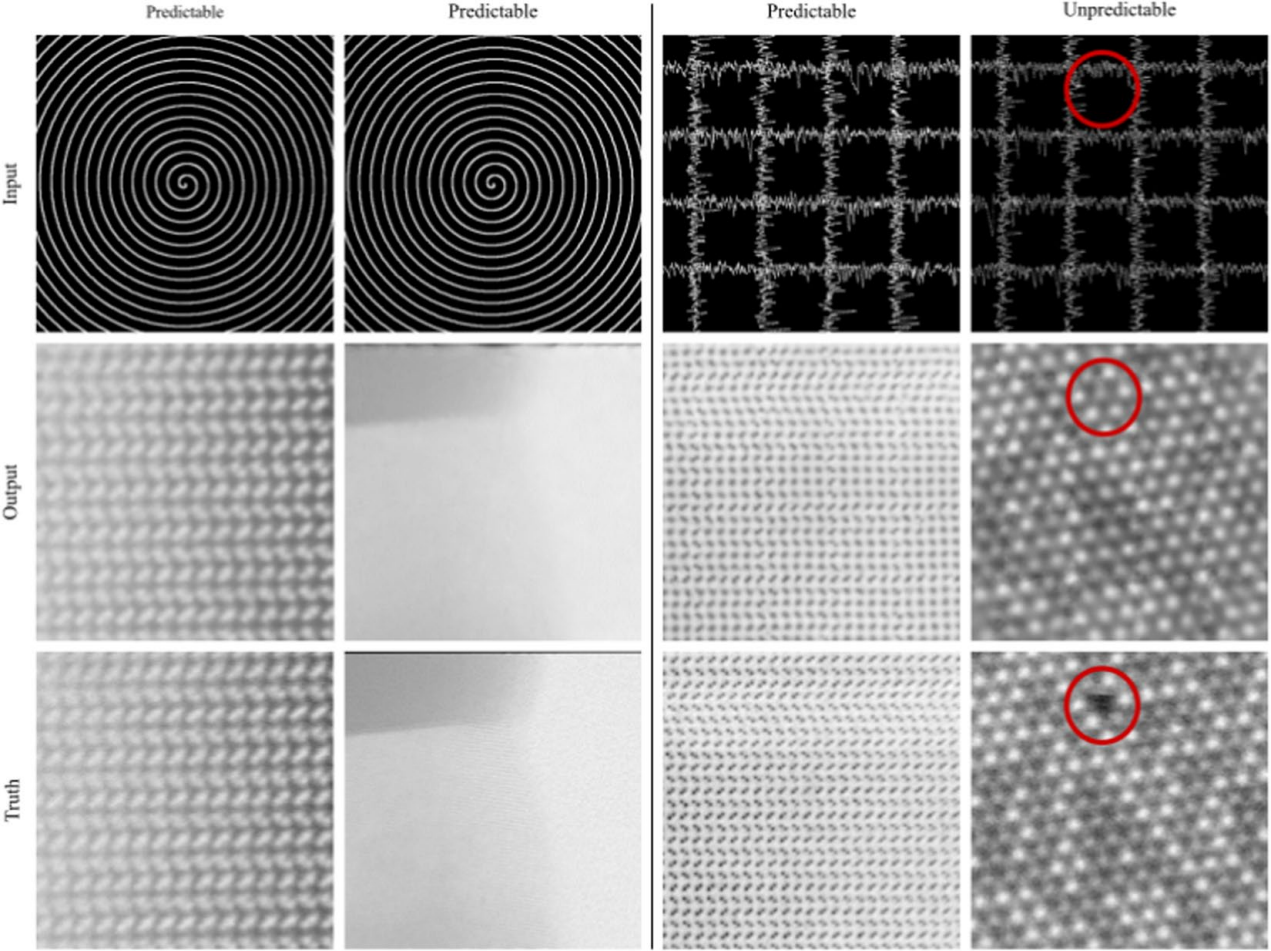
Non-adversarial generator outputs for 512 × 512 1/20 px coverage blurred spiral and gridlike scan inputs. Images with predictable patterns or structure are accurately completed. Circles accentuate that generators cannot reliably complete unpredictable images where there is no information. This figure was created with Inkscape^83^.

In practice, 1/20 px scan coverage is sufficient to complete most spiral scans. However, generators cannot reliably complete micrographs with unpredictable structure in regions where there is no coverage. This is demonstrated by example applications of non-adversarial generators to 1/20 px coverage spiral and gridlike partial scans in Fig. 4. Most noticeably, a generator invents a missing atom at a gap in gridlike scan coverage. Spiral scans have lower errors than gridlike scans as spirals have smaller gaps between coverage. Additional sheets of examples for spiral scans selected with binary masks are provided for scan coverages between 1/17.9 px and 1/87.0 px as Supplementary Information.

**Figure 5:**
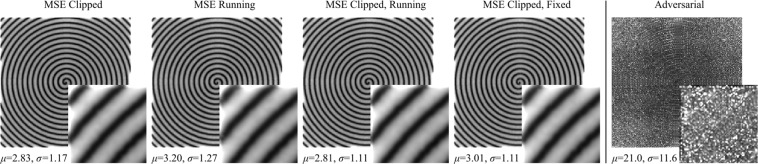
Generator mean squared errors (MSEs) at each output pixel for 20000 512 × 512 1/20 px coverage test set images. Systematic errors are lower near spiral paths for variants of MSE training, and are less structured for adversarial training. Means, *μ*, and standard deviations, *σ*, of all pixels in each image are much higher for adversarial outputs. Enlarged 64 × 64 regions from the top left of each image are inset to ease comparison, and to show that systematic errors for MSE training are higher near output edges.

To characterize generator performance, MSEs for output pixels are shown in Fig. 5. Errors were calculated for 20000 test set 1/20 px coverage spiral scans selected with blurred masks. Errors systematically increase with increasing distance from paths for non-adversarial training, and are less structured for adversarial training. Similar to other generators^23,64^, errors are also higher near the edges of non-adversarial outputs where there is less information. We tried various approaches to decrease non-adversarial systematic error variation by modifying loss functions. For examples: by ALRC; multiplying pixel losses by their running means; by ALRC and multiplying pixel losses by their running means; and by ALRC and multiplying pixel losses by final mean losses of a trained network. However, we found that systematic errors are similar for all variants. This is a limitation of partial STEM as information decreases with increasing distance from scan paths. Adversarial completions also exhibit systematic errors that vary with distance from spiral paths. However, spiral variation is dominated by other, less structured, spatial error variation. Errors are higher for adversarial training than for non-adversarial training as GANs complete images with realistic noise characteristics.

**Figure 6:**
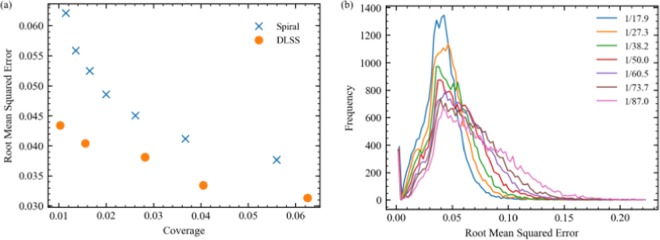
Test set root mean squared (RMS) intensity errors for spiral scans in $[0,1]$ selected with binary masks. (**a**) RMS errors decrease with increasing electron probe coverage, and are higher than deep learning supersampling^23^ (DLSS) errors. (**b**) Frequency distributions of 20000 test set RMS errors for 100 bins in $[0,0.224]$ and scan coverages in the legend.

Spiral path test set intensity errors are shown in Fig. 6a, and decrease with increasing coverage for binary masks. Test set errors are also presented for deep learning supersampling^23^ (DLSS) as they are the only results that are directly comparable. DLSS is an alternative approach to compressed sensing where STEM images are completed from a sublattice of probing locations. Both DLSS and partial STEM results are for the same neural network architecture, learning policy and training dataset. Results depend on datasets, so using the same dataset is essential for quantitative comparison. We find that DLSS errors are lower than spiral errors at all coverages. In addition, spiral errors exponentially increase above DLSS errors at low coverages where minimum distances from spiral paths increase. Although this comparison may appear unfavourable for partial STEM, we expect that this is a limitation of training signals being imaged at several times their Nyquist rates.

Distributions of 20000 spiral path test set root mean squared (RMS) intensity errors for spiral data in Fig. 6a are shown in Fig. 6b. The coverages listed in Fig. 6 are for infinite spiral paths with 1/16, 1/25, 1/36, 1/49, 1/64, 1/81, and 1/100 px coverage after paths are cut by image boundaries; changing coverage. All distributions have a similar peak near an RMS error of 0.04, suggesting that generator performance remains similar for a portion of images as coverage is varied. As coverage decreases, the portion of errors above the peak increases as generators have difficulty with more images. In addition, there is a small peak close to zero for blank or otherwise trivial completions.

## Discussion

Partial STEM can decrease scan coverage and total electron electron dose by 10–100× with 3–6% test set RMS errors. These errors are small compared to typical STEM noise. Decreased electron dose will enable new STEM applications to beam-sensitive materials, including organic crystals^65^, metal-organic frameworks^66^, nanotubes^67^, and nanoparticle dispersions^68^. Partial STEM can also decrease scan times in proportion to decreased coverage. This will enable increased temporal resolution of dynamic materials, including polar nanoregions in relaxor ferroelectrics^69,70^, atom motion^71^, nanoparticle nucleation^72^, and material interface dynamics^73^. In addition, faster scans can reduce delay for experimenters, decreasing microscope time. Partial STEM can also be a starting point for algorithms that process STEM images e.g. to find and interpret atomic positions^74^.

Our generators are trained for fixed coverages and 512 × 512 inputs. However, recent research has introduced loss function modifications that can be used to train a single generator for multiple coverages with minimal performance loss^23^. Using a single GAN improves portability as each of our GANs requires 1.3 GB of storage space with 32 bit model parameters, and limits technical debt that may accompany a large number of models. Although our generator input sizes are fixed, they can be tiled across larger images; potentially processing tiles in a single batch for computational efficiency. To reduce higher errors at the edge of generator outputs, tiles can be overlapped so that edges may be discarded^64^. Smaller images could be padded. Alternatively, dedicated generators can be trained for other output sizes.

There is an effectively infinite number of possible partial scan paths for 512 × 512 STEM images. In this paper, we focus on spiral and gridlike partial scans. For a fixed coverage, we find that the most effective method to decrease errors is to minimize maximum distances from input information. The less information there is about an output region, the more information that needs to be extrapolated, and the higher the error. For example, we find that errors are lower for spiral scans than gridlike scans as maximum distances from input information are lower. Really, the optimal scan shape is not static: It is specific to a given image and generator architecture. As a result, we are actively developing an intelligent partial scan system that adapts to inputs as they are scanned.

Partial STEM has a number of limitations relative to DLSS. For a start, partial STEM may require a custom scan system. Even if a scan system supports or can be reprogrammed to support custom scan paths, it may be insufficiently responsive. In contrast, DLSS can be applied as a postprocessing step without hardware modification. Another limitation of partial STEM is that errors increase with increasing distance from scan paths. Distances from continuous scan paths cannot be decreased without increasing coverage. Finally, most features in our new STEM crops dataset are sampled at several times their Nyquist rates. Electron microscopists often record images above minimum sufficient resolutions and intensities to ease visual inspection and limit the effects of drift^75^, shot^17^, and other noise. This means that a DLSS lattice can still access most high frequency information in our dataset.

Test set DLSS errors are lower than partial STEM errors for the same architecture and learning policy. However, this is not conclusive as generators were trained for a few days; rather than until validation errors diverged from training errors. For example, we expect that spirals need more training iterations than DLSS as nearest neighbour infilled spiral regions have varying shapes, whereas infilled regions of DLSS grids are square. In addition, limited high frequency information in training data limits one of the key strengths of partial STEM that DLSS lacks: access to high-frequency information from neighbouring pixels. As a result, we expect that partial STEM performance would be higher for signals imaged closer to their Nyquist rates.

To generate realistic images, we fine-tuned partial STEM generators as part of GANs. GANs generate images with more realistic high-frequency spatial components and structure than MSE training. However, GANs focus on semantics; rather than intensity differences. This means that although adversarial completions have realistic characteristics, such as high-frequency noise, individual pixel values differ from true values. GANs can also be difficult to train^76,77^, and training requires additional computation. Nevertheless, inference time is the same for adversarial and non-adversarial generators after training.

Encouragingly, ANNs are universal approximators^78^ that can represent^79^ the optimal mapping from partial scans with arbitrary accuracy. This overcomes the limitations of traditional algorithms where performance is fixed. If ANN performance is insufficient or surpassed by another method, training or development can be continued to achieve higher performance. Indeed, validation errors did not diverge from training errors during our experiments, so we are presenting lower bounds for performance. In this paper, we compare spiral STEM performance against DLSS. It is the only method that we can rigorously and quantitatively compare against as it used the same test set data. This yielded a new insight into how signals being imaged above their Nyquist rates may affect performance discussed two paragraphs earlier, and highlights the importance of standardized datasets like our new STEM images dataset. As machine learning becomes more established in the electron microscopy community, we hope that standardized datasets will also become established to standardize performance benchmarks.

Detailed neural network architecture, learning policy, experiments, and additional sheets of examples are provided as Supplementary Information. Further improvements might be made with AdaNet^80^, Ludwig^81^, or other automatic machine learning^82^ algorithms, and we encourage further development. In this spirit, we have made our source code^36^, a new dataset containing 16227 STEM images^40,41^, and pre-trained models publicly available. For convenience, new datasets containing 161069 non-overlapping 512 × 512 crops from STEM images used for training, and 19769 antialiased 96 × 96 area downsampled STEM images created for faster ANN development, are also available.

## Conclusions

Partial STEM with deep learning can decrease electron dose and scan time by over an order of magnitude with minimal information loss. In addition, realistic STEM images can be completed by fine-tuning generators as part of a GAN. Detailed MSE characteristics are provided for multiple coverages, including MSEs per output pixel for 1/20 px coverage spiral scans. Partial STEM will enable new beam sensitive applications, so we have made our source code, new STEM dataset, pre-trained models, and details of experiments available to encourage further investigation. High performance is achieved by the introduction of an auxiliary trainer network, and adaptive learning rate clipping of high losses. We expect our results to be generalizable to SEM and other scan systems.

## Supplementary information

Supplementary Information.

## Data Availability

New STEM datasets are available on our publicly accessible dataserver^40,41^. Source code for ANNs and to create images is in a GitHub repository with links to pre-trained models^36^. For additional information contact the corresponding author (J.M.E.).
