# Supplementary material for: Partial Scanning Transmission Electron Microscopy with Deep Learning
Source: Sci Rep. 2020 May 20;10:8332. doi: 10.1038/s41598-020-65261-0 (PMC7239858; doi:10.1038/s41598-020-65261-0)
Supplement: Supplementary file 1 — Supplementary Information. [file 41598_2020_65261_MOESM1_ESM.pdf]

Supplementary Information: Partial Scanning Transmission Electron Microscopy with Deep Learning

Jeffrey M. Ede[1,*] **and Richard Beanland**[1]

[1]University of Warwick, Department of Physics, Coventry, CV4 7AL, UK
[*]j.m.ede@warwick.ac.uk

S1 Detailed Architecture

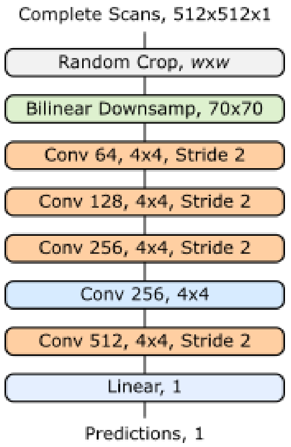

Figure S1. Discriminators examine random $w \times w$ crops to predict whether complete scans are real or generated. Generators are trained by multiple discriminators with different w. This figure was created with Inkscape[1].

Discriminator architecture is shown in Fig. S1. Generator and inner generator trainer architecture is shown in Fig. S2. The components in our networks are

Bilinear Downsamp, *w*x*w*: This is an extension of linear interpolation in one dimension to two dimensions. It is used to downsample images to $w \times w$.

Bilinear Upsamp, x*s*: This is an extension of linear interpolation in one dimension to two dimensions. It is used to upsample images by a factor of s.

Conv *d*, *w*x*w*, Stride, *x*: Convolutional layer with a square kernel of width, w, that outputs d feature channels. If the stride is specified, convolutions are only applied to every xth spatial element of their input, rather than to every element. Striding is not applied depthwise.

Linear, *d*: Flatten input and fully connect it to d feature channels.

Random Crop, *w*x*w*: Randomly sample a $w \times w$ spatial location using an external probability distribution.

⊕: Circled plus signs indicate residual connections where incoming tensors are added together. These help reduce signal attenuation and allow the network to learn perturbative transformations more easily.

All generator convolutions are followed by running mean-only batch normalization then ReLU activation, except output convolutions. All discriminator convolutions are followed by slope 0.2 leaky ReLU activation.

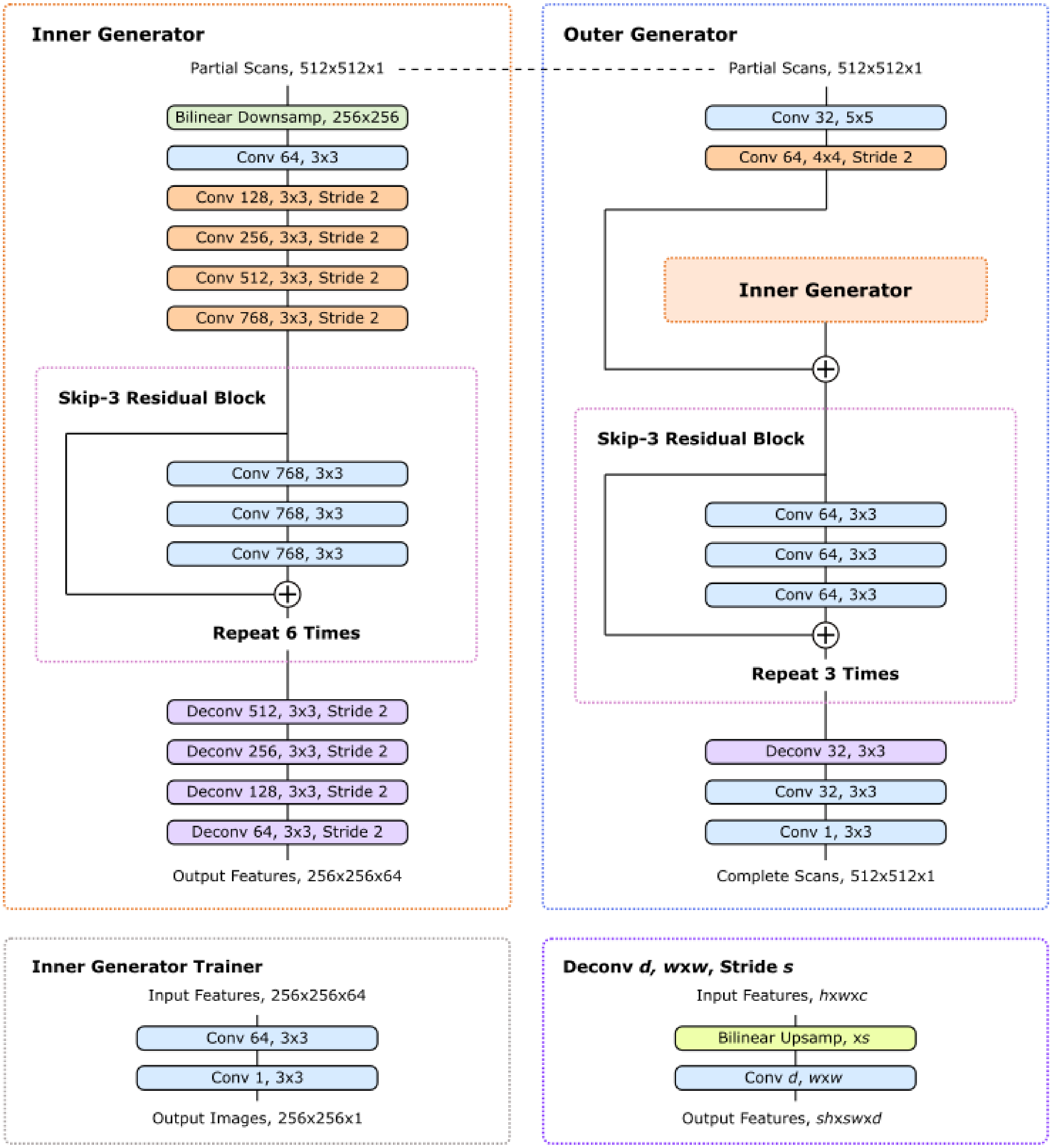

Figure S2. Two-stage generator that completes 512×512 micrographs from partial scans. A dashed line indicates that the same image is input to the inner and outer generator. Large scale features developed by the inner generator are locally enhanced by the outer generator and turned into images. An auxiliary trainer network restores images from inner generator features to provide direct feedback. This figure was created with Inkscape[1].

S2 Learning Policy

Optimizer: Training is ADAM[2] optimized and has two halves. In the first half, the generator and auxiliary trainer learn to minimize mean squared errors between their outputs and ground truth images. For the quarter of iterations, we use a constant learning rate $\eta_0 = 0.0003$ and a decay rate for the first moment of the momentum $\beta_1 = 0.9$. The learning rate is then stepwise decayed to zero in eight steps over the second quarter of iterations. Similarly, β_1 is stepwise linearly decayed to 0.5 in eight steps. In an optional second half, the generator and discriminators play an adversarial game conditioned on MSE guidance. For the third quarter of iterations, we use $\eta = 0.0001$ and $\beta_1 = 0.9$ for the generator and discriminators. In the final quarter of iterations, the generator learning rate is decayed to zero in eight steps while the discriminator learning rate remains constant. Similarly, generator and discriminator β_1 is stepwise decayed to 0.5 in eight steps.

Experiments with GAN training hyperparameters show that $\beta_1 = 0.5$ is a good choice[3]. Our decision to start at $\beta_1 = 0.9$ aims to improve the initial rate of convergence. In the first stage, generator and auxiliary trainer parameters are both updated once per training step. In the second stage, all parameters are updated once per training step. In most of our initial experiments with burred masks, we used a total of 10^6 training iterations. However, we found that validation errors do not diverge if training time is increased to 2×10^6 iterations, and used this number for experiments with binary masks. These training iterations are in-line with with other GANs, which reuse datasets containing a few thousand examples for 200 epochs[4]. The lack of validation divergence suggests that performance may be substantially improved, and means that our results present lower bounds for performance. All training was performed with a batch size of 1 due to the large model size needed to complete 512×512 scans.

Adaptive learning rate clipping: To stabilize batch size 1 training, adaptive learning rate clipping[5] (ALRC) was developed to limit high MSEs. ALRC layers were initialized with first raw moment $\mu_1 = 25$, second raw moment $\mu_2 = 30$, exponential decay rates $\beta_1 = \beta_2 = 0.999$, and $n = 3$ standard deviations.

Input normalization: Partial scans, I_{scan}, input to the generator are linearly transformed to $I'_{\text{scan}} = (I_{\text{scan}} + 1)/2$, where $I'_{\text{scan}} \in [0, 1]$. The generator is trained to output ground truth crops in $[0, 1]$, which are linearly transformed to $[-1, 1]$. Generator outputs and ground truth crops in $[-1, 1]$ are directly input to discriminators.

Weight normalization: All generator parameters are weight normalized[6]. Running mean-only batch normalization[6,7] is applied to the output channels of every convolutional layer, except the last. Channel means are tracked by exponential moving averages with decay rates of 0.99. Running mean-only batch normalization is frozen in the second half of training to improve stability[8].

Spectral normalization: Spectral normalization[3] is applied to the weights of each convolutional layer in the discriminators to limit the Lipschitz norms of the discriminators. We use the power iteration method with one iteration per training step to enforce a spectral norm of 1 for each weight matrix.

Spectral normalization stabilizes training, reduces susceptibility to mode collapse and is independent of rank, encouraging discriminators to use more input features to inform decisions[3]. In contrast, weight normalization[6] and Wasserstein weight clipping[9] impose more arbitrary model distributions that may only partially match the target distribution.

Activation: In the generator, ReLU[10] non-linearities are applied after running mean-only batch normalization. In the discriminators, slope 0.2 leaky ReLU[11] non-linearities are applied after every convolutional layer. Rectifier leakage encourages discriminators to use more features to inform decisions. Our choice of generator and discriminator non-linearities follows recent work on high-resolution conditional GANs[4].

Initialization: Generator weights were initialized from a normal distribution with mean 0.00 and standard deviation 0.05. To apply weight normalization, an example scan is then propagated through the network. Each layer output is divided by its L2 norm and the layer weights assigned their division by the square root of the L2 normalized output's standard deviation. There are no biases in the generator as running mean-only batch normalization would allow biases to grow unbounded c.f. batch normalization[12].

Discriminator weights were initialized from a normal distribution with mean 0.00 and standard deviation 0.03. Discriminator biases were zero initialized.

Experience replay: To reduce destabilizing discriminator oscillations[13], we used an experience replay[14,15] with 50 examples. Prioritizing the replay of difficult examples can improve learning[16], so we only replayed examples with losses in the top 20%. Training examples had a 20% chance to be sampled from the replay.

S3 Experiments

In this section, we present learning curves for some of our non-adversarial architecture and learning policy experiments. During training, each training set example was reused ~8 times. In comparison, some generative adversarial networks (GANs) are trained on the same data hundreds of times[4]. As a result, we did not experience noticeable overfitting. In cases where final

errors are similar; so that their difference is not significant within the error of a single experiment, we choose the lowest error approach. In practice, choices between similar errors are unlikely to have a substantial effect on performance. Each experiment took a few days with an Nvidia GTX 1080 Ti GPU. All learning curves are 2500 iteration boxcar averaged. In addition, the first 10^4 iterations before dashed lines in figures, where losses rapidly decrease, are not shown.

Following previous work on high-resolution GANs[4], we used a multi-stage training protocol for our initial experiments. The outer generator was trained separately; after the inner generator, before fine-tuning the inner and outer generator together. An alternative approach uses an auxiliary loss network for end-to-end training, similar to Inception[17,18]. This can provide a more direct path for gradients to back-propagate to the start of the network and introduces an additional regularization mechanism. Experimenting, we connected an auxiliary trainer to the inner generator and trained the network in a single stage. As shown by Fig. S3a, auxiliary network supported end-to-end training is more stable and converges to lower errors.

In encoder-decoders, residual connections[19] between strided convolutions and symmetric strided transpositional convolutions can be used to reduce information loss. This is common in noise removal networks where the output is similar to the input[20,21]. However, symmetric residual connections are also used in encoder-decoder networks for semantic image segmentation[22] where the input and output are different. Consequently, we tried adding symmetric residual connections between strided and transpositional inner generator convolutions. As shown by Fig. S3b, extra residuals accelerate initial inner generator training. However, final errors are slightly higher and initial inner generator training converged to similar errors with and without symmetric residuals. Taken together, this suggests that symmetric residuals initially accelerate training by enabling the final inner generator layers to generate crude outputs though their direct connections to the first inner generator layers. However, the symmetric connections also provide a direct path for low-information outputs of the first layers to get to the final layers, obscuring the contribution of the inner generator's skip-3 residual blocks (section S1) and lowering performance in the final stages of training.

Path information is concatenated to the partial scan input to the generator. In principle, the generator can infer electron beam paths from partial scans. However, the input signal is attenuated as it travels through the network[23]. In addition, path information would have to be deduced; rather than informing calculations in the first inner generator layers, decreasing efficiency. To compensate, paths used to generate partial scans from full scans are concatenated to inputs. As shown by Fig. S3b, concatenating path information reduces errors throughout training. Performance might be further improved by explicitly building sparsity into the network[24].

Large convolutional kernels are often used at the start of neural networks to increase their receptive field. This allows their first convolutions to be used more efficiently. The receptive field can also be increased by increasing network depth, which could also enable more efficient representation of some functions[25]. However, increasing network depth can also increase information loss[23] and representation efficiency may not be limiting. As shown by Fig. S3c, errors are lower for small first convolution kernels; 3×3 for the inner generator and 7×7 for the outer generator or both 3×3, than for large first convolution kernels; 7×7 for the inner generator and 17×17 for the outer generator. This suggests that the generator does not make effective use of the larger 17×17 kernel receptive field and that the variability of the extra kernel parameters harms learning.

Learning curves for different learning rate schedules are shown in Fig. S3d. Increasing training iterations and doubling the learning rate from 0.0002 to 0.0004 lowers errors. Validation errors do not plateau for 10^6 iterations in Fig. S3e, suggesting that continued training would improve performance. In our experiments, validation errors were calculated after every 50 training iterations.

The choice of output domain can affect performance. Training with a [0, 1] output domain is compared against $[-1,1]$ for slope 0.01 leaky ReLU activation after every generator convolution in Fig. S3f. Although $[-1,1]$ is supported by leaky ReLUs, requiring orders of magnitude differences in scale for $[-1,0)$ and $(0,1]$ hinders learning. To decrease dependence on the choice output domain, we do not apply batch normalization or activation after the last generator convolutions in our final architecture.

The $[0,1]$ outputs of Fig. S3f were linearly transformed to $[-1,1]$ and passed through a $\tanh$ non-linearity. This ensured that $[0,1]$ output errors were on the same scale as $[-1,1]$ output errors, maintaining the same effective learning rate. Initially, outputs were clipped by a $\tanh$ non-linearity to limit outputs far from the target domain from perturbing training. However, Fig. S4a shows that errors are similar without end non-linearites so they were removed. Fig. S4a also shows that replacing slope 0.01 leaky ReLUs with ReLUs and changing all kernel sizes to 3×3 has little effect. Swapping to ReLUs and 3×3 kernels is therefore an option to reduce computation. Nevertheless, we continue to use larger kernels throughout as we think they would usefully increase the receptive field with more stable, larger batch size training.

To more efficiently use the first generator convolutions, we nearest neighbour infilled partial scans. As shown by Fig. S4b, infilling reduces error. However, infilling is expected to be of limited use for low-dose applications as scans can be noisy, making meaningful infilling difficult. Nevertheless, nearest neighbour partial scan infilling is a computationally inexpensive method to improve generator performance for high-dose applications.

To investigate our generator's ability to handle STEM noise[26], we combined uniform noise with partial scans of Gaussian blurred STEM images. More noise was added to low intensity path segments and low-intensity pixels. As shown by Fig. S4c,

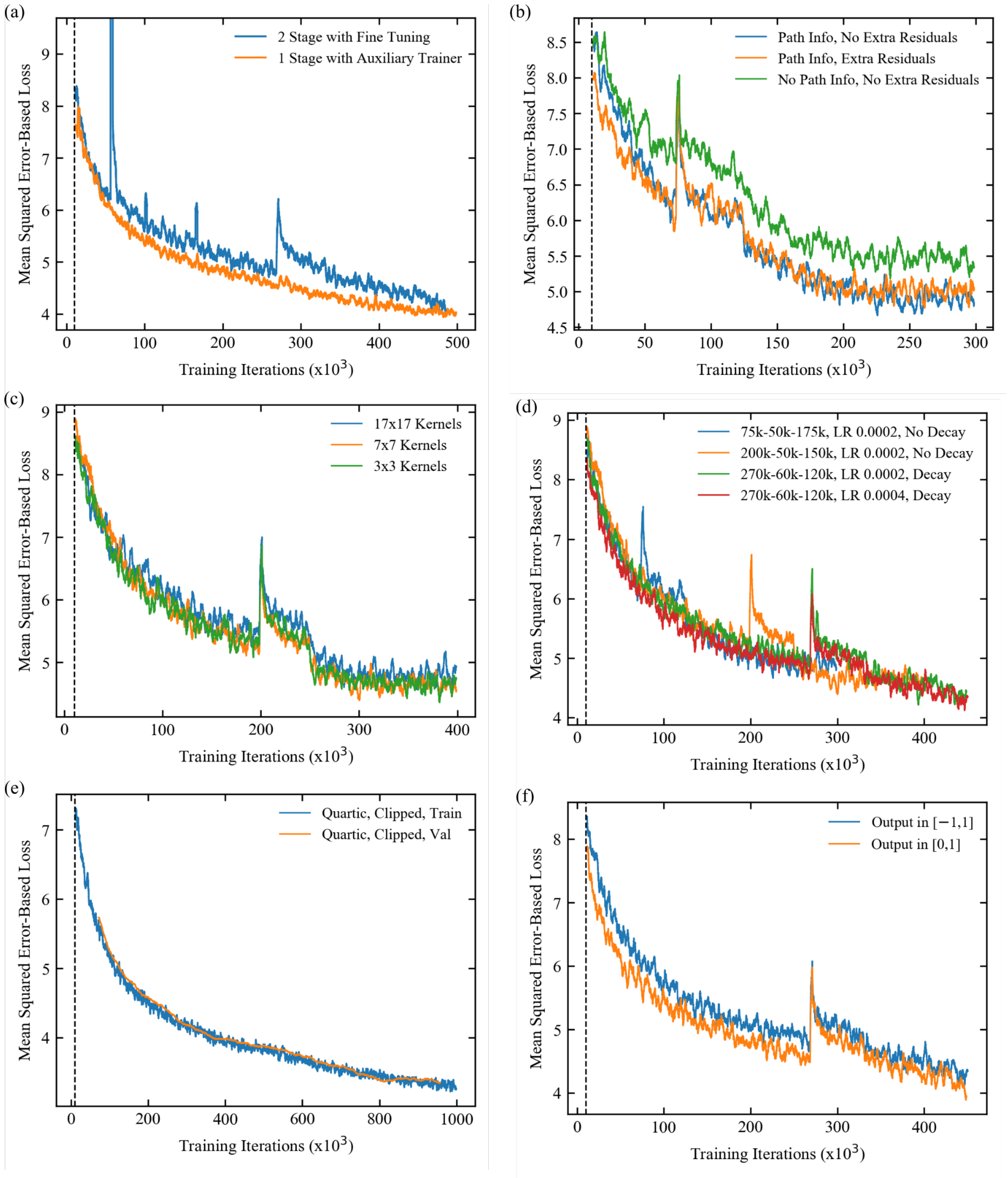

Figure S3. Learning curves. a) Training with an auxiliary inner generator trainer stabilizes training, and converges to lower than two-stage training with fine tuning. b) Concatenating beam path information to inputs decreases losses. Adding symmetric residual connections between strided inner generator convolutions and transpositional convolutions increases losses. c) Increasing sizes of the first inner and outer generator convolutional kernels does not decrease losses. d) Losses are lower after more interations, and a learning rate (LR) of 0.0004; rather than 0.0002. Labels indicate inner generator iterations - outer generator iterations - fine tuning iterations, and k denotes multiplication by 1000 e) Adaptive learning rate clipped quartic validation losses have not diverged from training losses after 10^6 iterations. f) Losses are lower for outputs in [0, 1] than for outputs in [-1, 1] if leaky ReLU activation is applied to generator outputs.

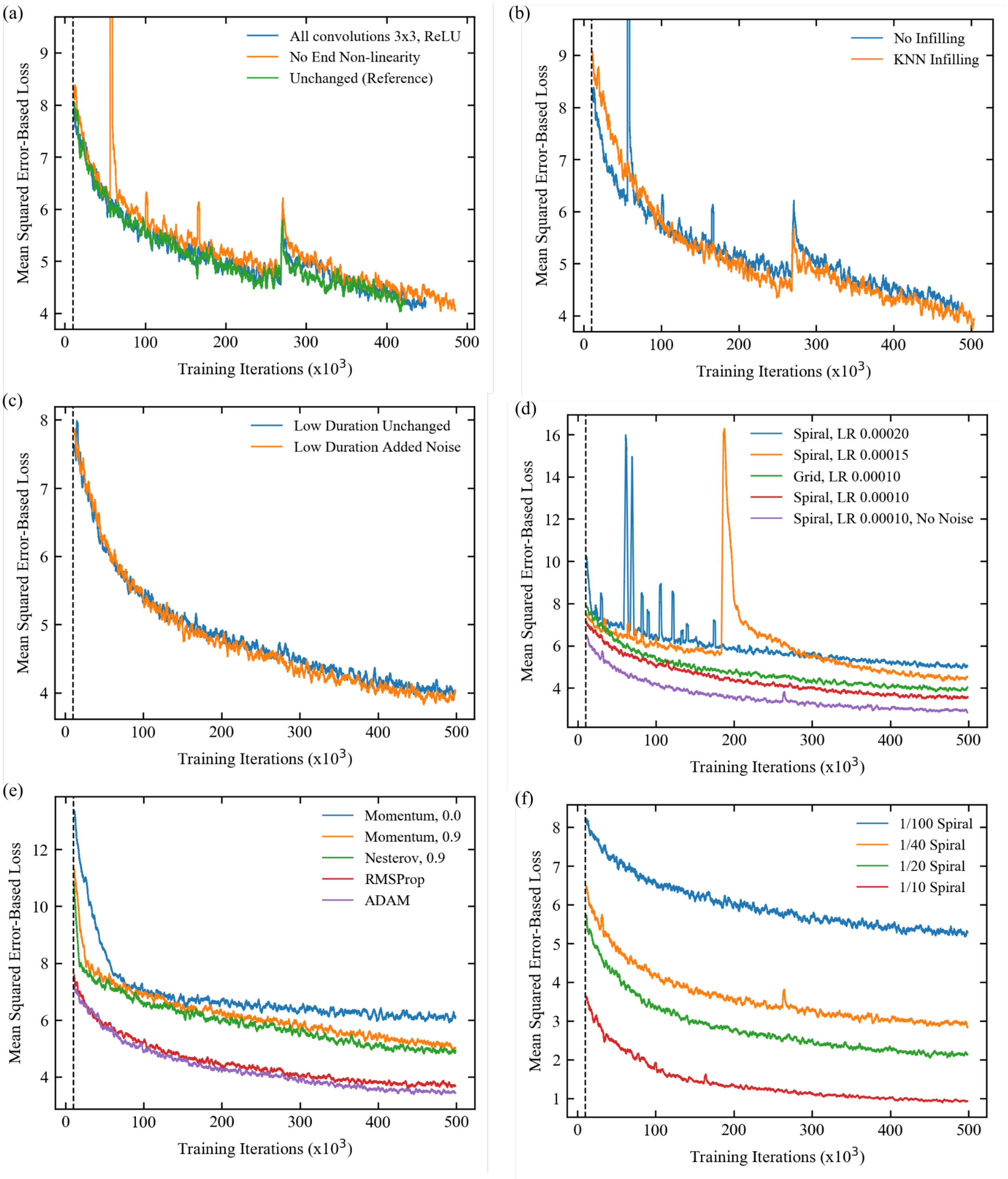

Figure S4. Learning curves. a) Making all convolutional kernels 3×3, and not applying leaky ReLU activation to generator outputs does not increase losses. b) Nearest neighbour infilling decreases losses. Noise was not added to low duration path segments for this experiment. c) Losses are similar whether or not extra noise is added to low-duration path segments. d) Learning is more stable and converges to lower errors at lower learning rates (LRs). Losses are lower for spirals than grid-like paths, and lowest when no noise is added to low-intensity path segments. e) Adaptive momentum-based optimizers, ADAM and RMSProp, outperform non-adaptive momentum optimizers, including Nesterov-accelerated momentum. ADAM outperforms RMSProp; however, training hyperparameters and learning protocols were tuned for ADAM. Momentum values were 0.9. f) Increasing partial scan pixel coverages listed in the legend decreases losses.

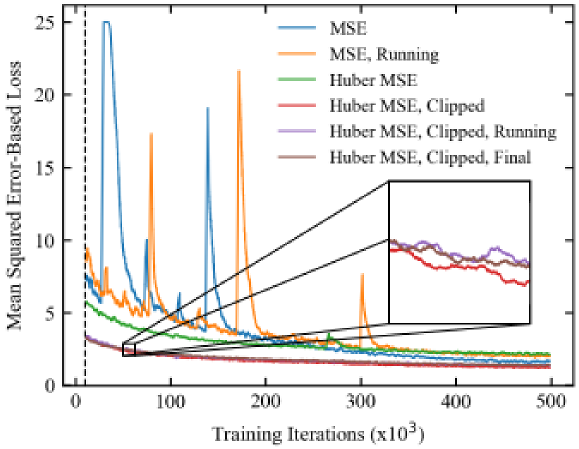

Figure S5. Adaptive learning rate clipping stabilizes learning, accelerates convergence and results in lower errors than Huberisation. Weighting pixel errors with their running or final mean errors is ineffective.

ablating extra noise for low-duration path segments increases performance.

Fig. S4d shows that spiral path training is more stable and reaches lower errors at lower learning rates. At the same learning rate, spiral paths converge to lower errors than grid-like paths as spirals have more uniform coverage. Errors are much lower for spiral paths when both intensity- and duration-dependent noise is ablated.

To choose a training optimizer, we completed training with stochastic gradient descent, momentum, Nesterov-accelerated momentum[27,28], RMSProp[29] and ADAM[2]. Learning curves are in Fig. S4e. Adaptive momentum optimizers, ADAM and RMSProp, outperform the non-adaptive optimizers. Non-adaptive momentum-based optimizers outperform momentumless stochastic gradient decent. ADAM slightly outperforms RMSProp; however, architecture and learning policy were tuned for ADAM. This suggests that RMSProp optimization may also be a good choice.

Learning curves for 1/10, 1/20, 1/40 and 1/100 px coverage spiral scans are shown in Fig. S4f. In practice, 1/20 px coverage is sufficient for most STEM images. On average, a non-adversarial generator can complete test set 1/20 px coverage partial scans with a 2.6% root mean squared intensity error. Nevertheless, higher coverage is needed to resolve fine detail in some images. Likewise, lower coverage may be appropriate for images without fine detail. Consequently, we are developing an intelligent scan system that adjusts coverage based on micrograph content.

Training is performed with a batch size of 1 due to the large network size needed for 512×512 partial scans. However, MSE training is unstable and large error spikes destabilize training. To stabilize learning, we developed adaptive learning rate clipping[5] (ALRC) to limit magnitudes of high losses while preserving their initial gradient distributions. ALRC is compared against MSE, Huberised MSE, and weighting each pixel's error by its Huberised running mean, and fixed final errors in Fig. S5. ALRC results in more stable training with the fastest convergence and lowest errors. Similar improvements have been confirmed for CIFAR-10 and STEM supersampling with ALRC[5].

S4 Additional Examples

Sheets of examples comparing non-adversarial generator outputs and true images are shown in Fig. S6-S12 for 512×512 spiral scans selected with binary masks. True images are blurred by a 5×5 symmetric Gaussian kernel with a 2.5 px standard deviation so that they are the same as the images that generators were trained output. Images are blurred to suppress high-frequency noise. Examples are presented for 1/17.9, 1/27.3, 1/38.2, 1/50.0, 1/60.5, 1/73.7, and 1/87.0 px coverage, in that order, so that higher errors become apparent for decreasing coverage with increasing page number. Quantitative performance characteristics for each generator are provided in the main article.

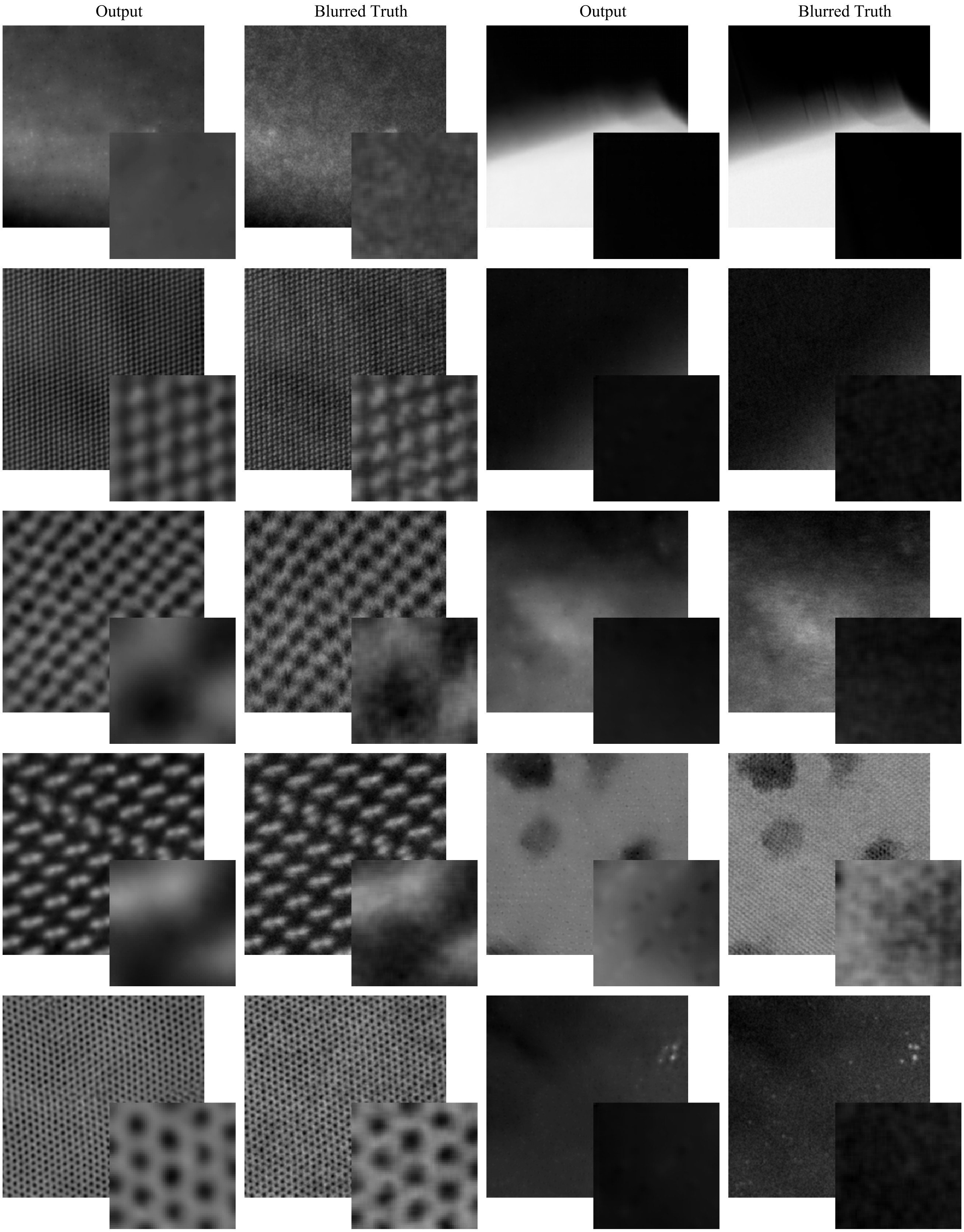

Figure S6. Non-adversarial 512×512 outputs and blurred true images for 1/17.9 px coverage spiral scans selected with binary masks.

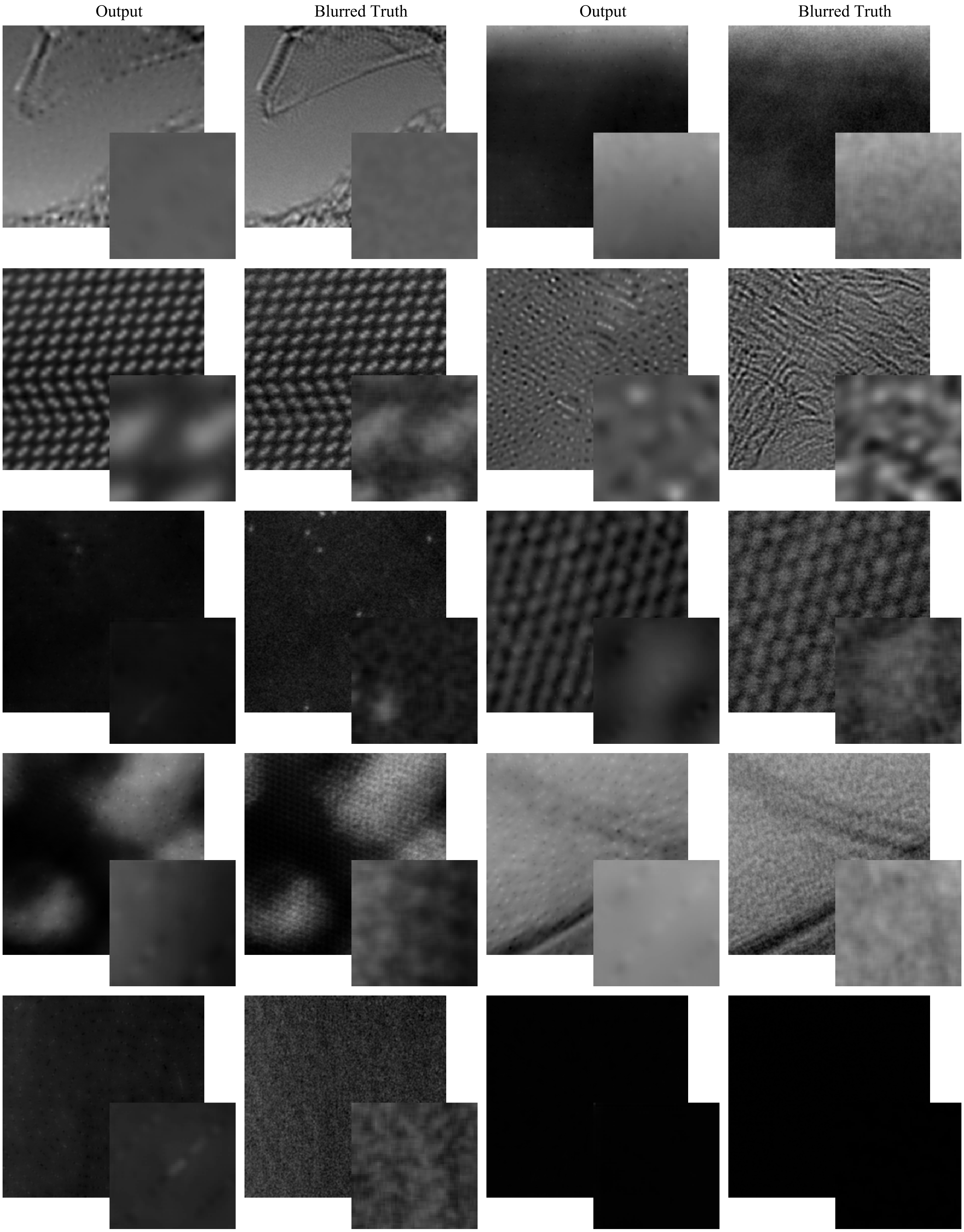

Figure S7. Non-adversarial 512×512 outputs and blurred true images for 1/27.3 px coverage spiral scans selected with binary masks.

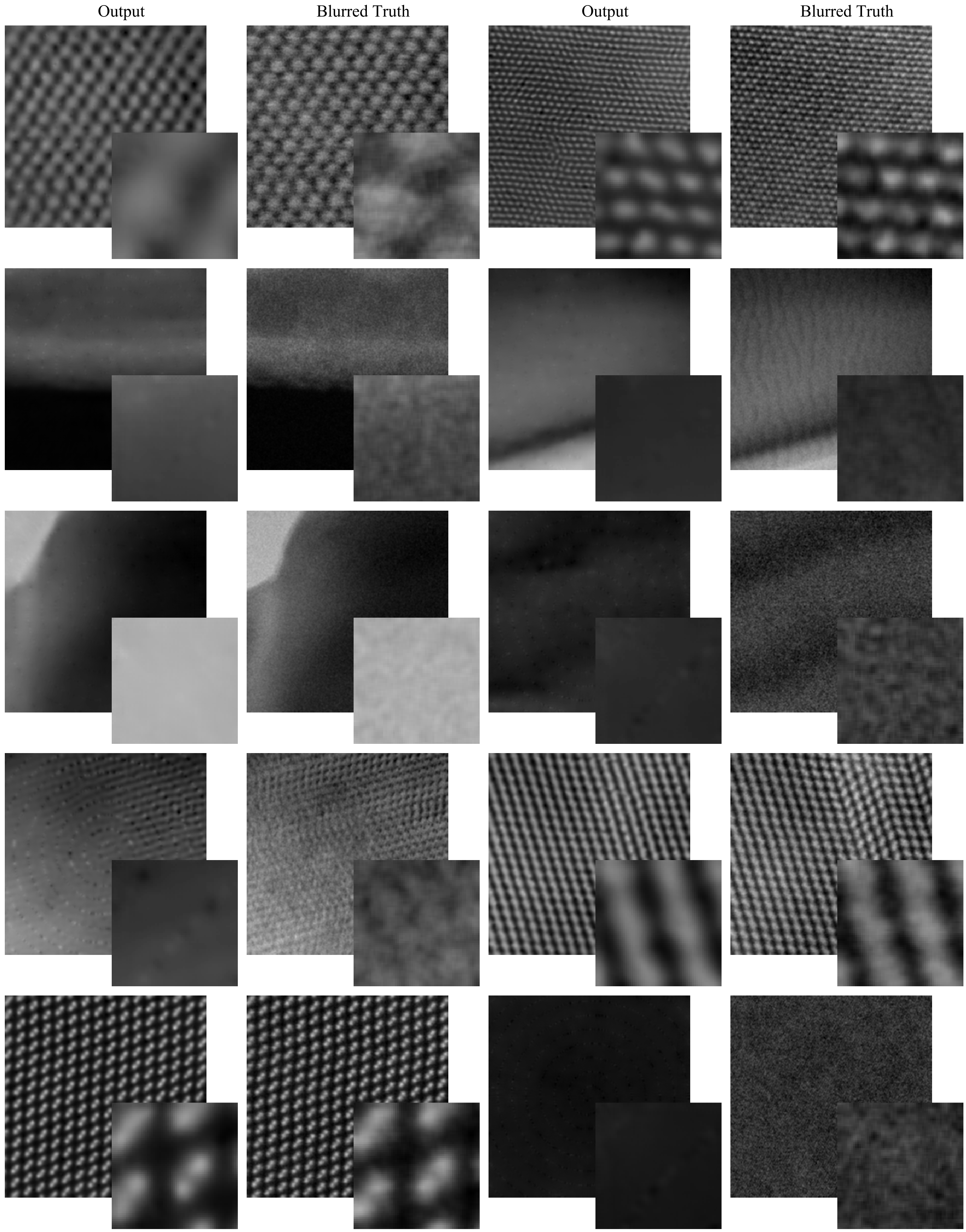

Figure S8. Non-adversarial 512×512 outputs and blurred true images for 1/38.2 px coverage spiral scans selected with binary masks.

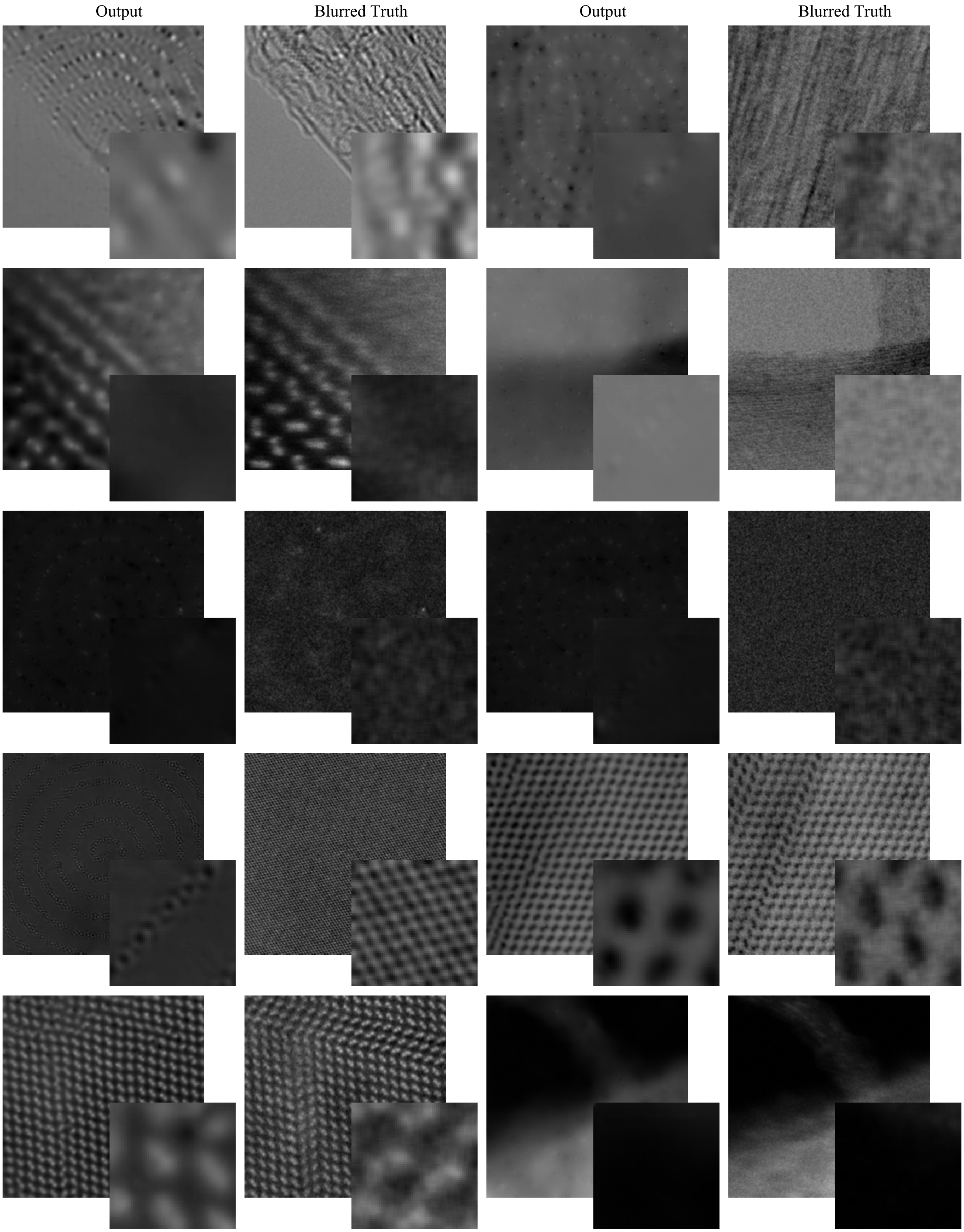

Figure S9. Non-adversarial 512×512 outputs and blurred true images for 1/50.0 px coverage spiral scans selected with binary masks.

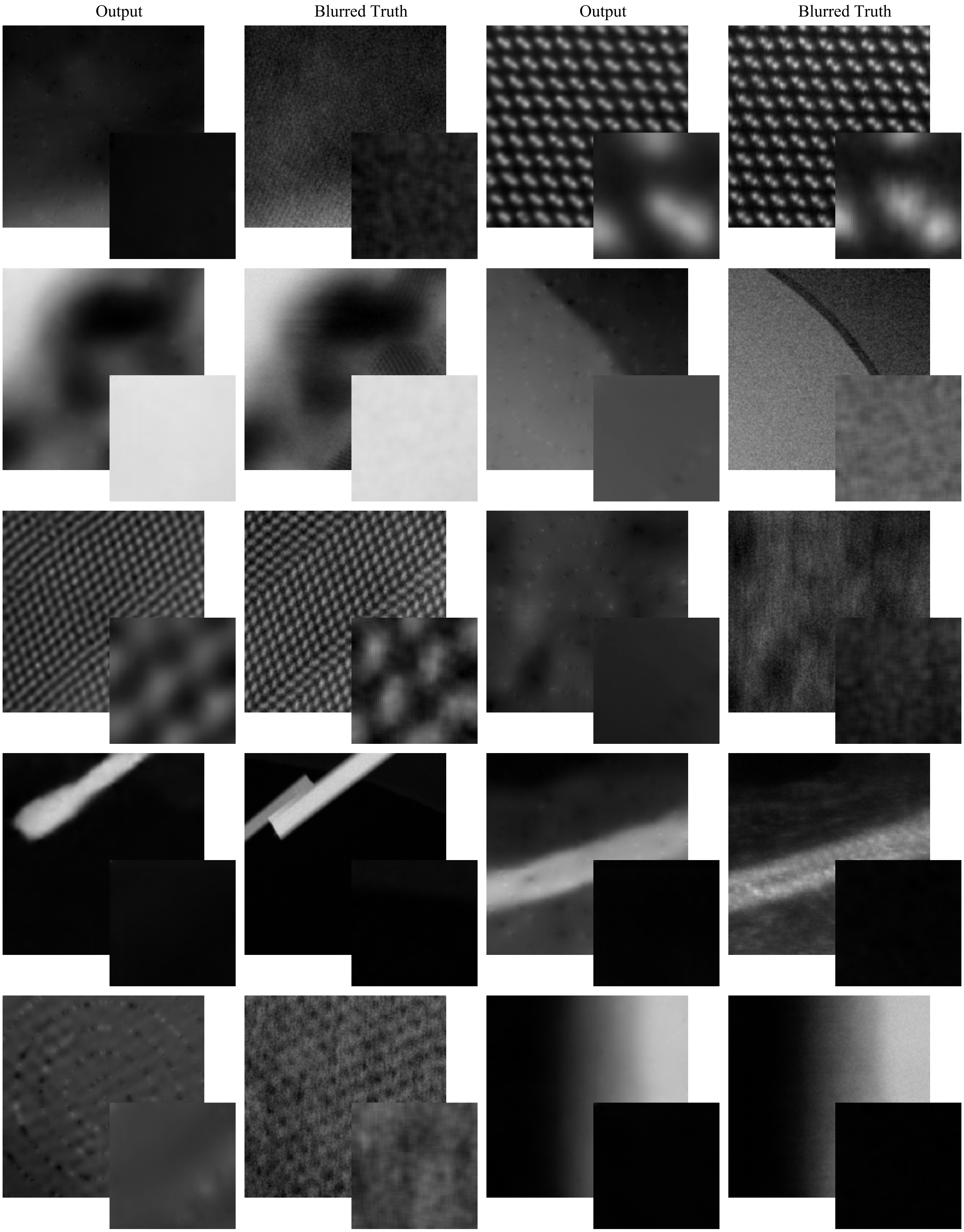

Figure S10. Non-adversarial 512×512 outputs and blurred true images for 1/60.5 px coverage spiral scans selected with binary masks.

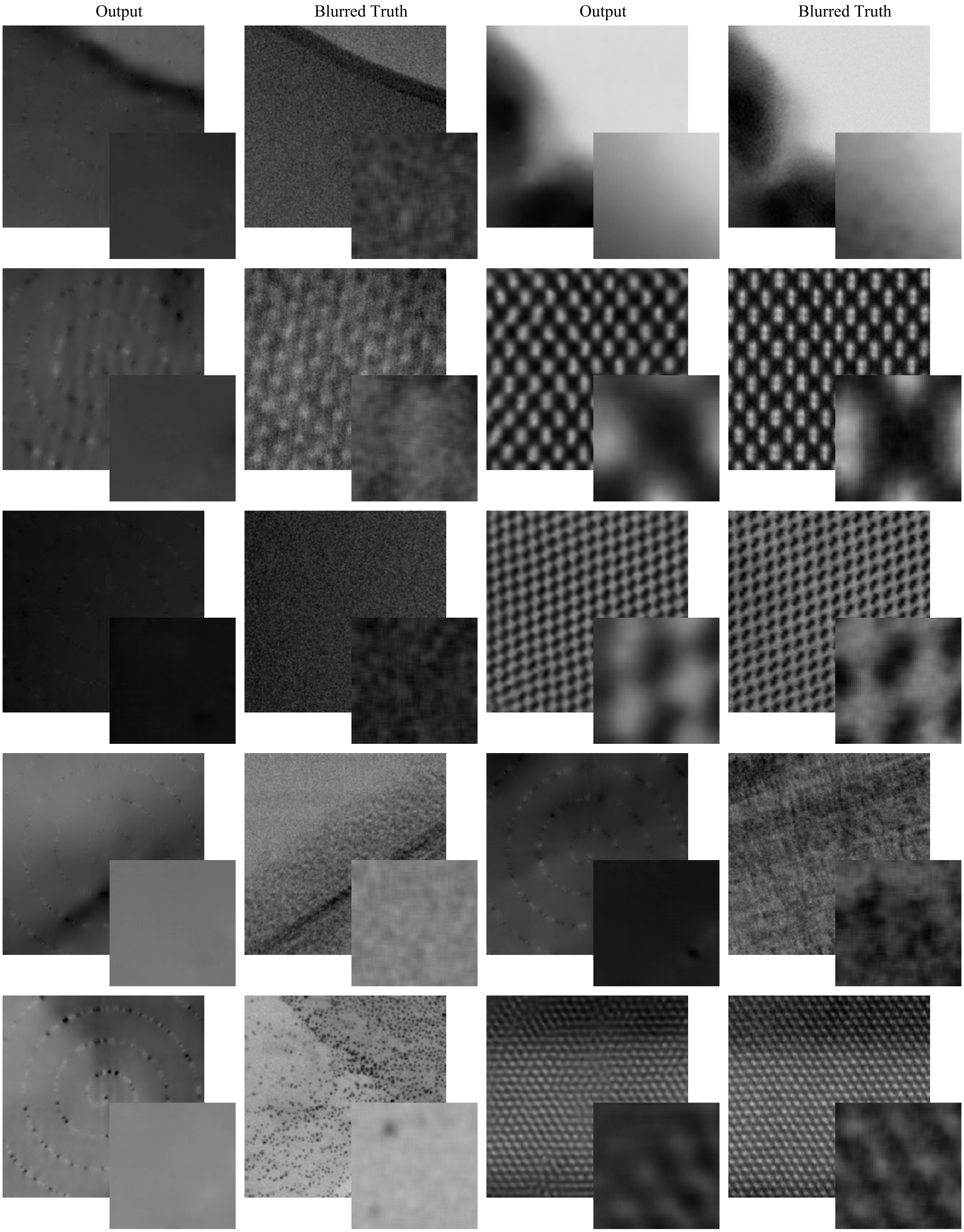

Figure S11. Non-adversarial 512×512 outputs and blurred true images for 1/73.7 px coverage spiral scans selected with binary masks.

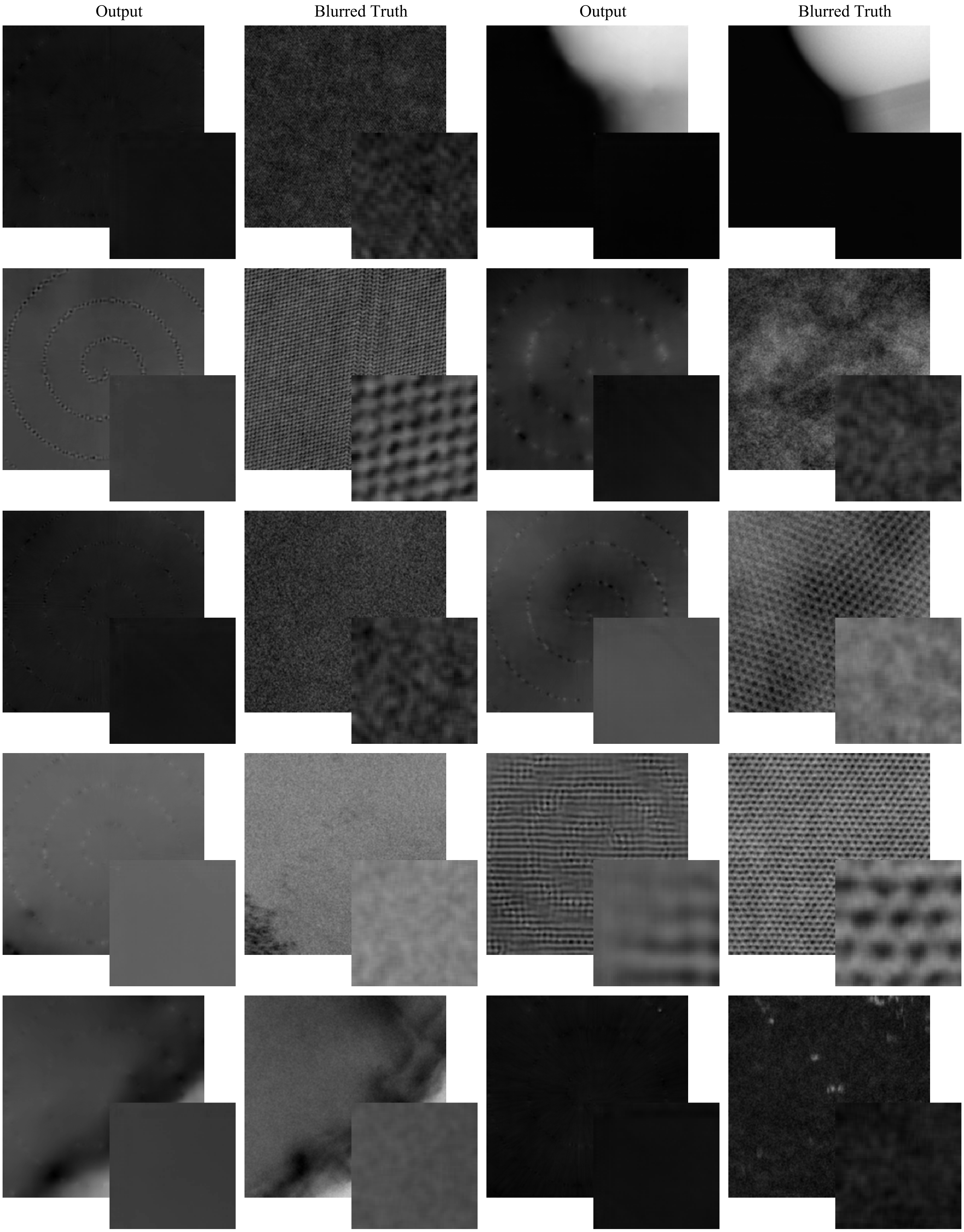

Figure S12. Non-adversarial 512×512 outputs and blurred true images for 1/87.0 px coverage spiral scans selected with binary masks.

References

1. Harrington, B. *et al.* Inkscape 0.92. Online: http://www.inkscape.org/ (2020).
2. Kingma, D. P. & Ba, J. ADAM: A Method for Stochastic Optimization. *arXiv preprint arXiv:1412.6980* (2014).
3. Miyato, T., Kataoka, T., Koyama, M. & Yoshida, Y. Spectral Normalization for Generative Adversarial Networks. *arXiv preprint arXiv:1802.05957* (2018).
4. Wang, T.-C. *et al.* High-Resolution Image Synthesis and Semantic Manipulation with Conditional GANs. In *Proceedings of the IEEE Conference on Computer Vision and Pattern Recognition*, 8798–8807 (2018).
5. Ede, J. M. & Beanland, R. Adaptive Learning Rate Clipping Stabilizes Learning. *Mach. Learn. Sci. Technol.* (2020).
6. Salimans, T. & Kingma, D. P. Weight Normalization: A Simple Reparameterization to Accelerate Training of Deep Neural Networks. In *Advances in Neural Information Processing Systems*, 901–909 (2016).
7. Hoffer, E., Banner, R., Golan, I. & Soudry, D. Norm Matters: Efficient and Accurate Normalization Schemes in Deep Networks. In *Advances in Neural Information Processing Systems*, 2160–2170 (2018).
8. Chen, L.-C., Papandreou, G., Schroff, F. & Adam, H. Rethinking Atrous Convolution for Semantic Image Segmentation. *arXiv preprint arXiv:1706.05587* (2017).
9. Arjovsky, M., Chintala, S. & Bottou, L. Wasserstein Generative Adversarial Networks. In *International Conference on Machine Learning*, 214–223 (2017).
10. Nair, V. & Hinton, G. E. Rectified Linear Units Improve Restricted Boltzmann Machines. In *Proceedings of the 27th International Conference on Machine Learning (ICML-10)*, 807–814 (2010).
11. Maas, A. L., Hannun, A. Y. & Ng, A. Y. Rectifier Nonlinearities Improve Neural Network Acoustic Models. In *Proceedings of the International Conference on Machine Learning*, vol. 30, 3 (2013).
12. Ioffe, S. & Szegedy, C. Batch Normalization: Accelerating Deep Network Training by Reducing Internal Covariate Shift. *arXiv preprint arXiv:1502.03167* (2015).
13. Liang, K. J., Li, C., Wang, G. & Carin, L. Generative Adversarial Network Training is a Continual Learning Problem. *arXiv preprint arXiv:1811.11083* (2018).
14. Pfau, D. & Vinyals, O. Connecting Generative Adversarial Networks and Actor-Critic Methods. *arXiv preprint arXiv:1610.01945* (2016).
15. Shrivastava, A. *et al.* Learning from Simulated and Unsupervised Images through Adversarial Training. *arXiv preprint arXiv: 161207828* (2016).
16. Schaul, T., Quan, J., Antonoglou, I. & Silver, D. Prioritized Experience Replay. *arXiv preprint arXiv:1511.05952* (2015).
17. Szegedy, C. *et al.* Going Deeper with Convolutions. In *Proceedings of the IEEE Conference on Computer Vision and Pattern Recognition*, 1–9 (2015).
18. Szegedy, C., Vanhoucke, V., Ioffe, S., Shlens, J. & Wojna, Z. Rethinking the Inception Architecture for Computer Vision. In *Proceedings of the IEEE conference on Computer Vision and Pattern Recognition*, 2818–2826 (2016).
19. He, K., Zhang, X., Ren, S. & Sun, J. Deep Residual Learning for Image Recognition. In *Proceedings of the IEEE Conference on Computer Vision and Pattern Recognition*, 770–778 (2016).
20. Mao, X.-J., Shen, C. & Yang, Y.-B. Image Restoration using Convolutional Auto-encoders with Symmetric Skip Connections. *arXiv preprint arXiv:1606.08921* (2016).
21. Casas, L., Navab, N. & Belagiannis, V. Adversarial Signal Denoising with Encoder-Decoder Networks. *arXiv preprint arXiv:1812.08555* (2018).
22. Badrinarayanan, V., Kendall, A. & Cipolla, R. SegNet: A Deep Convolutional Encoder-Decoder Architecture for Image Segmentation. *IEEE Transactions on Pattern Analysis Mach. Intell.* **39**, 2481–2495 (2017).
23. Zheng, H., Yao, J., Zhang, Y. & Tsang, I. W. Degeneration in VAE: In the Light of Fisher Information Loss. *arXiv preprint arXiv:1802.06677* (2018).
24. Graham, B. Spatially-Sparse Convolutional Neural Networks. *arXiv preprint arXiv:1409.6070* (2014).
25. Lin, H. W., Tegmark, M. & Rolnick, D. Why does Deep and Cheap Learning Work so Well? *J. Stat. Phys.* **168**, 1223–1247 (2017).

26. Seki, T., Ikuhara, Y. & Shibata, N. Theoretical Framework of Statistical Noise in Scanning Transmission Electron Microscopy. *Ultramicroscopy* **193**, 118–125 (2018).

27. Sutskever, I., Martens, J., Dahl, G. & Hinton, G. On the Importance of Initialization and Momentum in Deep Dearning. In *International Conference on Machine Learning*, 1139–1147 (2013).

28. Nesterov, Y. A Method of Solving a Convex Programming Problem with Convergence Rate O($1/k^2$). In *Soviet Mathematics Doklady*, vol. 27, 372–376 (1983).

29. Hinton, G., Srivastava, N. & Swersky, K. Neural Networks for Machine Learning Lecture 6a Overview of Mini-Batch Gradient Descent (2012).
